# Temporal Changes in the Hepatic Fatty Liver in Mice Receiving Standard Lieber-DeCarli Diet

**DOI:** 10.5487/TR.2008.24.2.113

**Published:** 2008-06-01

**Authors:** Hu-Quan Yin, Byung-Hoon Lee

**Affiliations:** College of Pharmacy and Research Institute of Pharmaceutical Sciences, Seoul National University, San 56-1, Sillim-dong, Gwanak-gu, Seoul, 151-742 Korea

**Keywords:** Ethanol, Lieber-DeCarli diet, Fatty liver, Triglyceride, Sterol regulatory element binding protein

## Abstract

Chronic exposure to ethanol induces cumulative damage to the liver starting from fatty infiltration to cirrhosis depending on the dose and duration of exposure. The whole process leading to the development of alcoholic liver disease is very complex and the mechanisms involved are not fully understood. Among many experimental animal models, Lieber-DeCarli liquid diet provides moderate to severe pathophysiological outcome depending on the compositional changes. In the present study, we investigated the temporal changes in the early phase hepatic disease in rats fed with standard Lieber-DeCarli diet. Male Wistar rats were fed with Lieber-Decarli ethanol diet for 6 weeks and the liver samples were obtained after 2, 4 and 6 weeks. Mild fatty infiltration was observed in 2 weeks of feeding and it became evident in 4 and 6 week samples. The level of hepatic triglyceride showed a good agreement with the data obtained in the pathological analysis. Feeding mice with ethanol diet resulted in the maturation and translocation of SREBP-1 to nucleus in the liver. Western blot analysis of the pooled liver sample of control and ethanol fed animals showed a clear-cut time-dependent increase in the expression of nSREBP-1. These data provide important information for selecting proper time point in experimental intervention study in the field of drug development for alcoholic liver disease.

## INTRODUCTION

Ethanol is one of the main causes of end-stage liver disease throughout the world (Mandayam et al, 2004). Chronic exposure to ethanol induces cumulative damage to the liver starting from fatty infiltration, inflammation and focal necrosis to terminal venular sclerosis that can ultimately develop into cirrhosis depending on the dose and duration of exposure. All these symptoms or pathophysiological states are termed collectively alcoholic liver disease (ALD). Because of the wide spectrum of the nature of the disease, the whole process leading to the development of ALD is very complex and the mechanisms involved are not fully understood.

Alcoholic steatosis, or fatty liver, is an early and reversible consequence of chronic and/or excessive alcohol consumption. Although it was formerly considered a benign and fully reversible condition, hepatic fatty infiltration, or steatosis, is an important pathogenic factor in the development of alcoholic liver disease. Therefore, there have been many attempts to develop effective therapeutics for the disease using drugs, chemicals and natural products derived from medicinal plants (Kang and Kim, 1995). Mechanisms underlying lipid accumulation in the liver caused by diverse classes of hepatotoxic chemicals include an oversupply of free fatty acids to the liver, increases in the hepatic fatty acid synthesis, and decrease in the fatty acid oxidation, apoprotein synthesis and secretion (Treinen-Moslen, 2001). Enhanced hepatic fatty acid synthesis and reduced fatty acid oxidation act together in the development of an alcoholic fatty liver (Feinman and Lieber, 1999; You *et al*., 2002; You and Crabb, 2004).

Various animal models have been developed to study alcoholic liver disease (Hall et *al*., 2001; Järveläinen and Lindros, 2002). Among them Lieber-DeCarli model is the one that mimics human pathophysiological situation, and provides technically simple way to reproduce early stages of alcoholic liver disease. It is therefore one of the most widely used model in the study of alcoholic liver disease. However the temporal progression of the disease in this model is not yet clear, which needs to be elucidated not only to understand the nature of the model but to determine the exact time point to start intervention. In the present study we report the temporal changes in the pathophysiology of the liver in animals receiving the standard Lieber-DeCarli liquid diet.

## MATERIALS AND METHODS

***Animal treatments.*** Specific pathogen-free male Wistar rats (180 g~200 g) were obtained from Jung-Ang Laboratory Animal Co. (Seoul, Korea) and allowed free access to standard chow and tap water during the acclimation period. They were kept in temperature-controlled and filter-sterilized animal quarters under a 12-h light : 12-h dark cycle. The use of animals was in compliance with the guidelines established by the Animal Care Committee of this institute. A total 16 mice (4 per group) were fed either Lieber-Decarli ethanol diet that 36% of total calories supplied from ethanol (ethanol group) or Lieber-DeCarli control diet (control group). Controls were pair fed an equicaloric amount of diet with ethanol replaced by maltodextrin (Lieber and Decarli, 1989). The composition of the diet is listed in Table 1. Mice from the ethanol groups were sacrificed 2, 4 and 6 weeks after ethanol feeding. A cross section of the left lateral lobe of the liver was collected in 10% neutral buffered formalin for histopathology.

**Table 1.** Composition of the standard Lieber-DeCarli diet

| Ingredient | Control diet (g/liter; 1000 kcal) | Ethanol diet (g/liter; 1000 kcal) |
|---|---|---|
| Casein (100 Mesh) | 41.4 | 41.4 |
| L-Cystine | 0.5 | 0.5 |
| DL-Methionine | 0.3 | 0.3 |
| Corn Oil | 8.5 | 8.5 |
| Olive Oil | 28.4 | 28.4 |
| Safflower Oil | 2.7 | 2.7 |
| Maltose Dextrin | 115.2 | 25.6 |
| Cellulose | 10 | 10 |
| Choline Bitartrate | 0.53 | 0.53 |
| Xanthan Gum | 3 | 3 |
| Salt Mix[a] | 8.75 | 8.75 |
| Vitamin Mix[b] | 2.5 | 2.5 |
| Ethanol | 0 | 48 |

[a]Salt Mix (g/kg mix): Calcium Phosphate, dibasic, 500; Sodium Chloride, 74; Potassium Citrate, monohydrate, 220; Potassium Sulfate, 52; Magnesium Oxide, 24; Manganous Sulfate $H_2O$, 4.6; Ferrous Sulfate $7H_2O$, 4.95; Zinc Carbonate, 1.6; Cupric Carbonate, 0.3; Potassium Iodate, 0.01; Sodium Selenite, 0.01; Chromium Potassium Sulfate, 0.55; Sodium Fluoride, 0.06; Sucrose, finely powdered, 117.92.
[b]Vitamin Mix (g/kg mix): Thiamin HCl, 0.6; Riboflavin, 0.6; Pyridoxine HCl, 0.7; Niacin, 3.0; Calcium Pantothenate, 1.6; Folic Acid, 0.2; Biotin, 0.02; Vitamin B12 (0.1%), 10; Vitamin A Acetate (500,000 IU/g), 4.8; Vitamin D3 (400,000 IU/g), 24; Menadione Sodium Bisulfite, 0.08; P-Amino Benzoic Acid, 5; Inositol, 10; Dextrose, 939.

***Clinical chemistry and histological analysis.*** Serum levels of alanine aminotransferase (ALT) and aspartate amino transferase (AST) were monitored by standard clinical chemistry assays on an Automated Chemistry Analyzer (Prestige 24I; Tokyo Boeki Medical System, Tokyo, Japan). Liver tissues collected in formalin were dehydrated, embedded in paraffin, sectioned at 4 μm, and stained with hematoxylin and eosin (H&E). Histopathologic examinations of the liver sections were conducted by a pathologist and were peer-reviewed.

***Determination of triglyceride.*** Total liver lipids were extracted from homogenate prepared from 100 mg of rat liver using chloroform/methanol mix (2 : 1, v/v) (Bligh and Dyer, 1956). Triglycerides in total lipid were determined enzymatically using a commercially available enzymatic kit (Sigma Chem. Co.) according to the manufacturer's protocol.

***Western blot analysis.*** Nuclear extracts from primary cultured rat hepatocytes were prepared using commercially available kit (BioVison, Mountain View, CA) according to the manufacturer's instructions. Forty micrograms of the protein were separated by 10% SDS-polyacrylamide gel electrophoresis, transferred to a nitrocellulose membrane and immunoblotted with rabbit polyclonal Srebf1 antibody (Abcam, Cambridge, MA). Detection was performed by using enhanced chemiluminescence Western Blotting Detection Reagents (Amersham, Piscataway, NJ).

***Statistical analysis.*** Data are given as mean and standard deviation and inter-group differences were analyzed using the Student's t-test.

## RESULTS

Male Wistar rats were fed the standard Lieber-DeCarli ethanol diet for 2, 4 and 6 weeks and hepatotoxicity was evaluated by clinical chemistry. Serum ALT level was elevated significantly in ethanol group compared to control group from the 4 weeks of treatment, which was increased until the end of the experiment. A significant increase in the serum AST level was observed only at 4 weeks after the start of the feeding (Fig. 1A). In the histological analysis, fatty infiltration was observed in all the ethanol fed groups. Mild fatty infiltration was observed in 2 weeks of feeding and it became evident

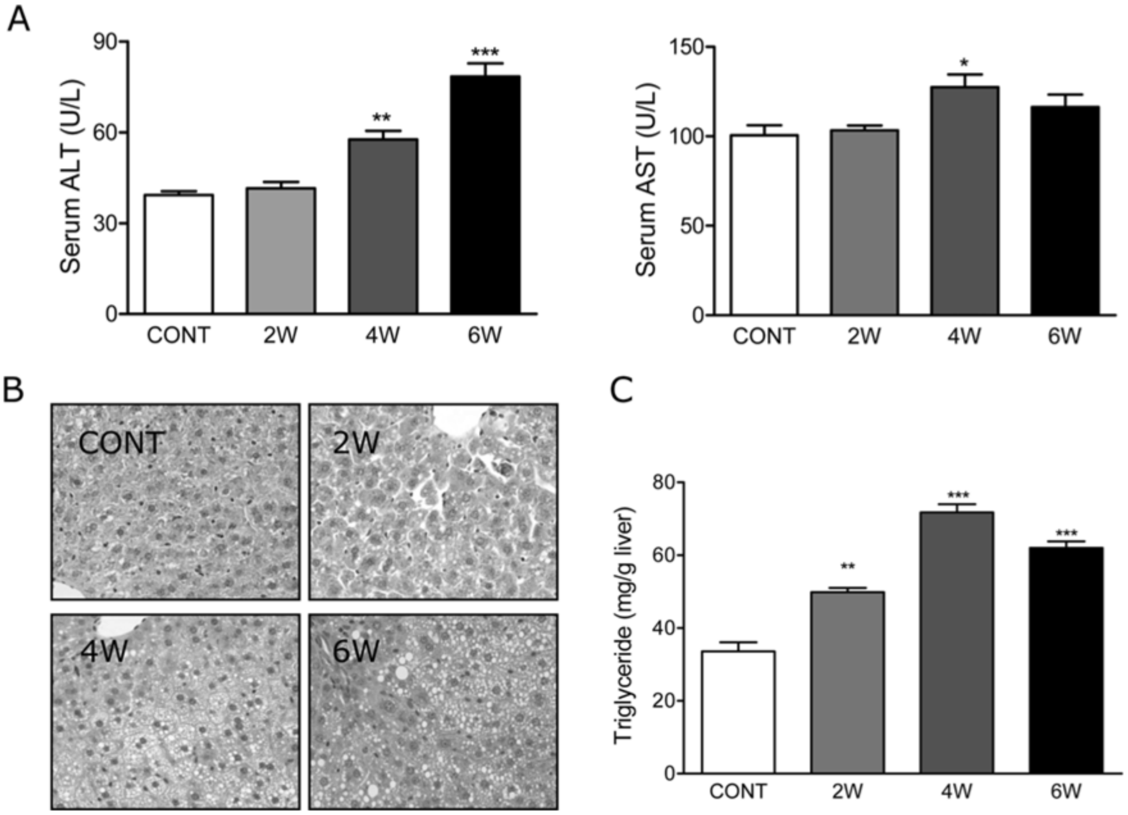

**Fig. 1.** Temporal changes in the development of fatty liver in ethanol fed rats. Male Wistar rats were fed Lieber-DeCarli ethanol diet and sacrificed after 2, 4 and 6 weeks. Serum ALT and AST levels were determined by standard clinical chemistry assays (A). Formalin fixed liver samples were processed, stained with hematoxylin and eosin and observed with light microscope (× 400) (B). Hepatic triglyceride level was measured by commercially available kit (*$p < 0.05$; **$p < 0.01$).

in 4 and 6 week samples (Fig. 1B). The level of hepatic triglyceride showed a very good agreement with the data obtained in the pathological analysis. Fatty liver is diagnosed clinically when lipid content in the liver exceeds 5~10% by weight (Adams *et al.*, 2005). In the 2 week ethanol fed animals hepatic triglyceride concentration was increased to 5.0 ± 0.3% of the total liver weight (Fig. 1C), which means that feeding ethanol for 2 weeks is enough to induce alcoholic fatty liver and that it is the proper time point for the start of intervention study. Triglyceride content was further increased to 7.2 ± 0.5% at 4 weeks but decreased slightly thereafter.

Sterol regulatory element-binding proteins (SREBPs) are a family of membrane-bound transcription factors that regulate lipid homeostasis by controlling the expression of genes for cholesterol and fatty acid metabolism. Chronic ethanol feeding induces the fatty acid synthesis pathway by the activation of SREBP-1, which contributes to the development of an alcoholic fatty liver (You *et al.*, 2002). Treatment of mice with Lieber-DeCarli ethanol diet resulted in the maturation and translocation of SREBP-1 to nucleus. Western blot analysis of the pooled liver sample of control and ethanol fed animals showed a clear-cut time-dependent increase

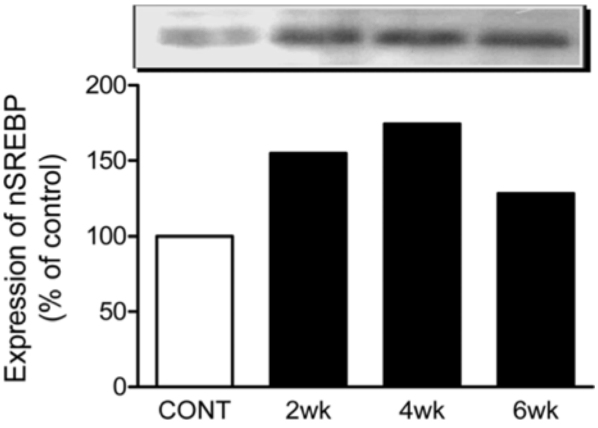

**Fig. 2.** Temporal changes in the expression of SREBP1 protein in the liver of ethanol fed rats. Aliquots of nuclear extracts obtained from pooled liver samples from 4 rats were electrophoresed on 10% SDS-polyacrylamide gel, transferred to a nitrocellulose membrane and immunoblotted with the rabbit polyclonal SREBP1 antibody.

in the expression of nSREBP-1 (Fig. 2).

## DISCUSSION

Steatosis, or fatty liver, is the earliest and mildest pathologic changes induced by alcohol consumption, drugs and abnormal metabolic states including disturbed triglyceride cycle and insulin resistance. Chronic consumption of ethanol causes cumulative liver damage ranging from steatosis to the end stage liver disease depending on the dose and duration of exposure. Although it was formerly considered a benign and fully reversible condition, hepatic fatty infiltration is an important pathogenic factor in the development of alcoholic liver disease. (Feinman and Lieber, 1999; You *et al*., 2002; You and Crabb, 2004).

There have been many attempts to develop effective therapeutics for the disease using drugs, chemicals and natural products derived from medicinal plants. Until now there exists no comprehensive animal model that could completely represent the human alcoholic liver disease (Hall *et al*., 2001). Therefore different models have been designed and tested to answer specific questions about different aspects of the disease (Nanji and French, 2003). Two animal models are used frequently to induce alcoholic liver disease, i.e. Tsukamoto-French intragastric feeding model and Lieber-DeCarli liquid diet model. In case of Tsukamoto-French model, ethanol in a liquid diet is infused through a catheter directly to the stomach. Therefore the blood level of ethanol can be adjusted depending on the purposed stage of liver disease from steatosis to cirrhosis. Major drawbacks of this model are that surgical operation is required and the mode of organ damage does not reflect alcohol's effect on the human liver. However, cirrhosis is achieved in a few months, whereas it takes many years to achieve cirrhosis in humans and in baboons fed ethanol orally (Tsukamoto *et al*., 1995). Lieber-DeCarli liquid diet model is the simplest method to provide ethanol chronically without aversion to alcohol. Despite blood ethanol level fluctuates, in part due to circadian rhythm, the pattern of alcohol consumption is clinically relevant and the alcohol intake is sufficient to cause significant liver damage such as steatosis (Lieber and DeCarli, 1994). Hall *et al*. (2001) reviewed models of alcoholic liver disease in rodents and concluded that the Lieber-DeCarli liquid diet for administering alcohol orally has provided an excellent means for reproducing the early lesions of ALD, which include steatosis and oxidative stress.

Lieber-DeCarli liquid diet model has long been used not only to study the pathophysiological characteristics of the disease but also to test potential candidate treatment options. However, characterization of the animal model especially in terms of the temporal progression of the disease is not clearly reported, which may provide valuable information in determining the starting point of the intervention study. In the present study, we investigated the time factors determining the state of the disease in Lieber-DeCarli ethanol diet model. According to our data, feeding rats with standard Lieber-DeCarli formular for 2 weeks is sufficient to induce significant steatosis. The degree of steatosis determined by hepatic triglyceride concentration reveals that the liver samples meet the criteria for clinical diagnosis for steatosis (Adams *et al*., 2005).

SREBPs are membrane-bound transcription factors that regulate lipid homeostasis by controlling the expression of genes for lipid metabolism. As can be seen in Fig. 1C and Fig. 2, the pattern of temporal change in hepatic triglyceride level agrees well with that in the expression of nSREBP-1. Recently, You *et al*. (2002) have reported that the metabolism of ethanol increased hepatic lipogenesis by activating SREBP-1, and that this effect of ethanol may contribute to the development of an alcoholic fatty liver. Our results confirm the previous data on the one hand and present a temporal progression of the alcoholic steatosis on the other hand. These data provide important information for selecting proper time point of an experimental intervention study in the field of drug development for alcoholic liver disease.

## ACKNOWLEDGMENTS

This work was supported by the Korea Research Foundation Grant (KRF-2004-005-E00093).
